# Supplementary material for: Safety and Immunogenicity of a Booster Vaccination by CoronaVac or BNT162b2 in Previously Two-Dose Inactivated Virus Vaccinated Individuals with Negative Neutralizing Antibody
Source: Vaccines (Basel). 2022 Apr 3;10(4):556. doi: 10.3390/vaccines10040556 (PMC9025305; doi:10.3390/vaccines10040556)
Supplement: Supplementary file 1 [file vaccines-10-00556-s001.zip › vaccines-1630281-supplementary.pdf]

Supplementary Table S1. Subgroup analysis of Quantitative IgG level against Spike protein and Neutralizing antibody (NAb) positivity in BBIBP-CoR V and CoronaVac primary vaccinated after CoronaVac or BNT162b2 booster.

	Booster vaccination	CoronaVac group (n=98)		P-value (within CoronaVac group)	BNT162b2 group (n=136)		P-value (within BNT162b2 group)
	1st & 2nd vaccination	BBIBP-CoR V (n=15)	CoronaVac (n=83)		BBIBP-CoR V (n=21)	CoronaVac (n=115)	
Antibody level, BAU/ml, median (IQR*)	Baseline	5.8 (2.3-8.5) n=15	14.2 (10.2-23.5) n=83	<.0001	3.8 (2.4-6.3) n=21	11.6 (7.2-18.5) n=115	<.0001
	Day 30	41.4 (18.4-329.7) n=12	149.7 (84.5-259.0) n=80	.0264	2029.9 (988.2-3817.5) n=20	2323.7 (1501.1-3676.7) n=110	.3662
	Day 90	28.1 (11.4-245.1) n=10	90.6 (46.5-160.7) n=67	.0602	576.0 (316.1-1986.4) n=17	1062.9 (633.5-1685.3) n=109	.1574
NAb positivity against wild type	Day 30	75% (9/12)	93% (74/80)	.0913	100% (20/20)	100% (110/110)	1
	Day 90	50% (5/10)	84% (56/67)	.0279	94% (16/17)	100% (109/109)	.1349
NAb positivity against Delta	Day 30	33% (4/12)	89% (71/80)	<.0001	95% (19/20)	100% (110/110)	.1538
	Day 90	30% (3/10)	76% (51/67)	.0062	94% (16/17)	97% (106/109)	.4442
NAb positivity against Omicron	Day 30	8% (1/12)	8% (6/80)	1	55% (11/20)	72% (79/110)	.1865
	Day 90	10% (1/10)	7% (5/67)	1	24% (4/17)	52% (57/109)	.0363

*Interquartile range

Median were tested using Mann-Whitney test (By Prism GraphPad Software)

p-values were tested using Fisher's exact test

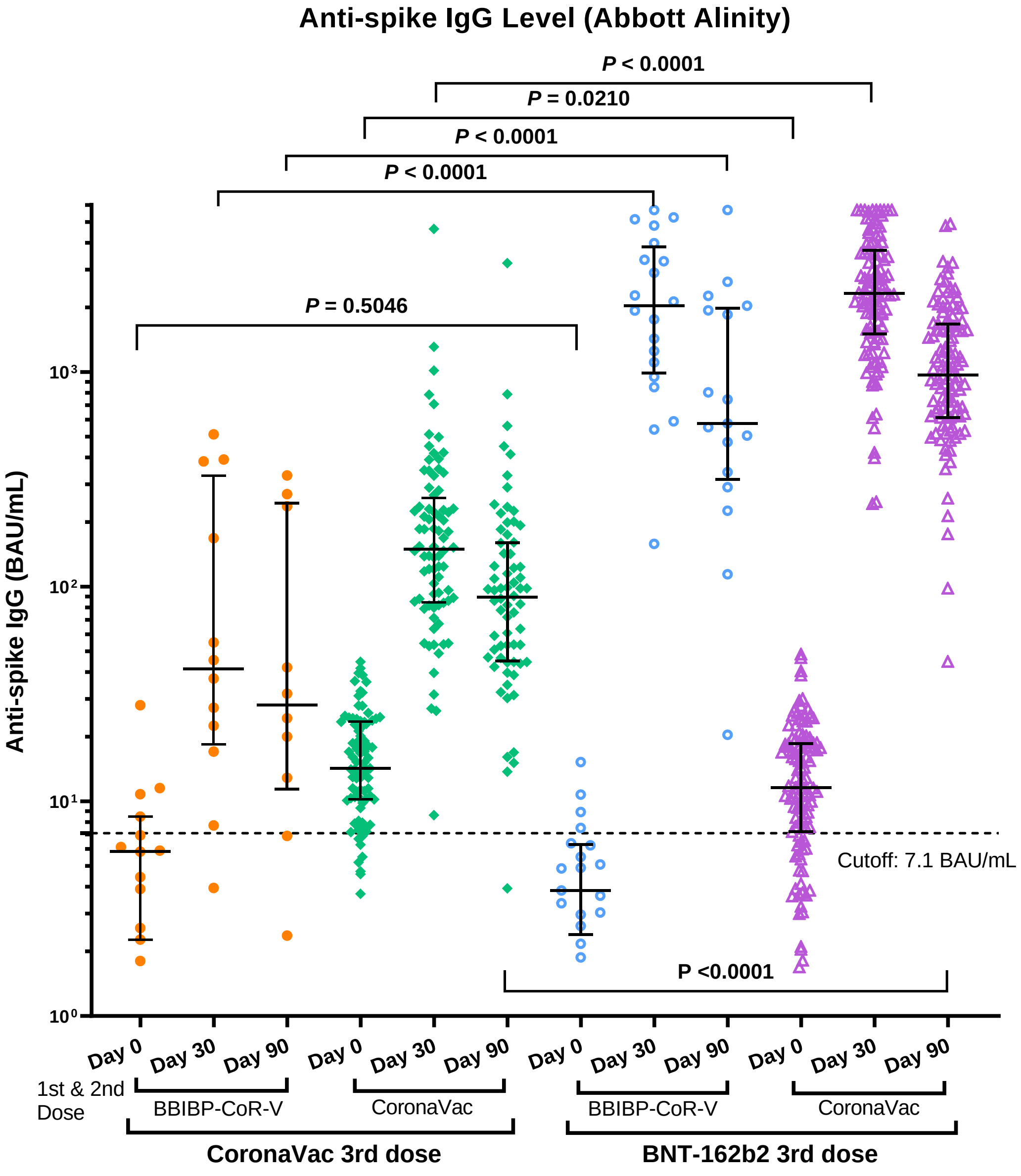

Anti-spike IgG Level (Abbott Alinity)
P < 0.0001
P = 0.0210
P < 0.0001
P < 0.0001
P = 0.5046
P <0.0001
Anti-spike IgG (BAU/mL)
10³
10²
10¹
10⁰
Cutoff: 7.1 BAU/mL
Day 0
Day 30
Day 90
Day 0
Day 30
Day 90
Day 0
Day 30
Day 90
Day 0
Day 30
Day 90
1st & 2nd Dose
BBIBP-CoR-V
CoronaVac
BBIBP-CoR-V
CoronaVac
CoronaVac 3rd dose
BNT-162b2 3rd dose

Figure S1 Comparison of immunogenicity of CoronaVac and BNT162b2 booster vaccination by quantitative anti-spike IgG in BBIBP-CoR V and CoronaVac primary vaccinated subgroups.

Supplementary Table S2. Time interval between 2nd dose and 3rd dose.

	Booster vaccination	CoronaVac group (n=98)			BNT162b2 group (n=136)			P-value (within CoronaVac)	P-Value (within BNT162b2)	P-Value (CoronaVac vs BNT162b2)
	Primary vaccination	BBIBP-CoR (n=15)	CoronaVac (n=83)	Overall	BBIBP-CoR (n=21)	CoronaVac (n=115)	Overall			
Interval (days) between 2nd & 3rd dose		210-221	94-215	94-221	205-265	124-222	124-265			
Median (days)		215	202	202	211	201	202	<.0001	<.0001	.8091
